# Effective/census population size ratio estimation: a compendium and appraisal

**DOI:** 10.1002/ece3.329

**Published:** 2012-07-25

**Authors:** Friso P Palstra, Dylan J Fraser

**Affiliations:** 1 CNRS UMR 7206 Eco-anthropologie et Ethnobiologie, Equipe “Génétique des populations humaines”, Muséum National d'Histoire NaturelleCP 139, 57 rue Cuvier, F-75231, Paris Cedex 05, France; 2 Department of Biology, Concordia University7141 Sherbrooke St. West, Montréal, Québec, Canada, H4B 1R6

**Keywords:** Conservation genetics, effective population size, empirical estimation, genetic stochasticity

## Abstract

With an ecological-evolutionary perspective increasingly applied toward the conservation and management of endangered or exploited species, the genetic estimation of effective population size (*N*_e_) has proliferated. Based on a comprehensive analysis of empirical literature from the past two decades, we asked: (i) how often do studies link *N*_e_ to the adult census population size (*N*)? (ii) To what extent is *N*_e_ correctly linked to *N*? (iii) How readily is uncertainty accounted for in both *N*_e_ and *N* when quantifying *N*_e_/*N* ratios? and (iv) how frequently and to what degree might errors in the estimation of *N*_e_ or *N* affect inferences of *N*_e_/*N* ratios? We found that only 20% of available *N*_e_ estimates (508 of 2617; 233 studies) explicitly attempted to link *N*_e_ and *N*; of these, only 31% (160 of 508) correctly linked *N*_e_ and *N*. Moreover, only 7% (41 of 508) of *N*_e_/*N* ratios (correctly linked or not) reported confidence intervals for both *N*_e_ and *N*; for those cases where confidence intervals were reported for *N*_e_ only, 31% of *N*_e_/*N* ratios overlapped with 1, of which more than half also reached below *N*_e_/*N* = 0.01. Uncertainty in *N*_e_*/N* ratios thus sometimes spanned at least two orders of magnitude. We conclude that the estimation of *N*_e_/*N* ratios in natural populations could be significantly improved, discuss several options for doing so, and briefly outline some future research directions.

## Background: why are effective and census population sizes important?

In many research instances in ecology and evolution, two important variables to be estimated in natural populations are the effective population size (*N*_e_) and the adult census size (*N*). As species ranges and abundances are continuously fragmented and/or reduced by human-induced environmental change, *N*_e_ and *N* will play key roles in determining the degree to which populations can avoid extinction from demographically, environmentally, or genetically stochastic events, such as temporary recruitment failures, environmental catastrophes, inbreeding depression, or a loss of genetic diversity at low population size (Soulé 1987; Boyce 1992; Frankham et al. 2003). Effective population size may also dictate whether populations can maintain adequate genetic variance for adaptive evolution in quantitative traits, and hence will affect responses to environmental change (Franklin 1980; Lynch and Lande 1997; Newman and Pilson 1997).

Knowledge of the relative magnitudes of these two parameters, as expressed by the ratio *N*_e_/*N*, is important for disentangling the relative risks that demographic, environmental, and genetic factors might pose for population persistence, particularly because *N*_e_ is generally much lower than *N* in natural populations (Frankham 1995; Palstra and Ruzzante 2008). Understanding *N*_e_/*N* ratios may also reveal what ecological factors drive *N*_e_ below *N*, insights which might facilitate more effective conservation and management decision-making (Kalinowski and Waples 2002). If simple conversions exist between *N*_e_ and *N* among taxonomic groups or intraspecific populations, much time and money could also be saved on the estimation of one variable to infer both (Luikart et al. 2010). Yet, several recent studies have suggested that no simple relationship between *N*_e_ and *N* may exist, either because of habitat factors or because of population expansion and contraction (Ardren and Kapuscinski 2003; Watts et al. 2007; Fraser et al. 2007b; Belmar-Lucero et al. 2012).

The precision and accuracy of various *N* estimators have seen extensive evaluation over the past century (Ricker 1975; Seber 1982; Pollack et al. 1990); so too has the estimation of *N*_e_ from genetic data in recent years, based on theoretical considerations (England et al. 2006; Waples and Do 2008), simulations (Jorde and Ryman 2007; Waples and Yokota 2007), and empirical data (Fraser et al. 2007a; Palstra and Ruzzante 2008). Now is the time to assess (i) the extent to which studies have linked *N*_e_ to the adult census population size (*N*); (ii) the extent to which *N*_e_ has been correctly linked to *N*; (iii) the degree to which uncertainty is accounted for in both *N*_e_ and *N* when quantifying *N*_e_/*N* ratios; and (iv) the frequency with which errors in the estimation of *N*_e_ or *N* affect inferences of *N*_e_/*N* ratios. These objectives form the present quantitative review, which considers the state of the field of empirical *N*_e_/*N* estimation and its future**.** Indeed, Frankham (2010) recently highlighted the updating of meta-analyses of *N*_e_/*N* ratios in the wild as a top priority scientific need in conservation genetics.

## Trends in published empirical estimates

### Trends in *N*_e_ estimation

We extended a previously compiled database of empirical estimates on contemporary *N*_e_ within natural populations based on genetic data (Palstra and Ruzzante 2008). New estimates were added through literature searches performed in ISI Web of Science (up to April 30th 2012), using the search terms “effective population size” and “microsatellites” or “allozymes”, and by performing queries on citations of key articles, usually on the methodology of estimating contemporary *N*_e_. We also browsed the Online Early sections of many relevant journals.

**Figure 1:**
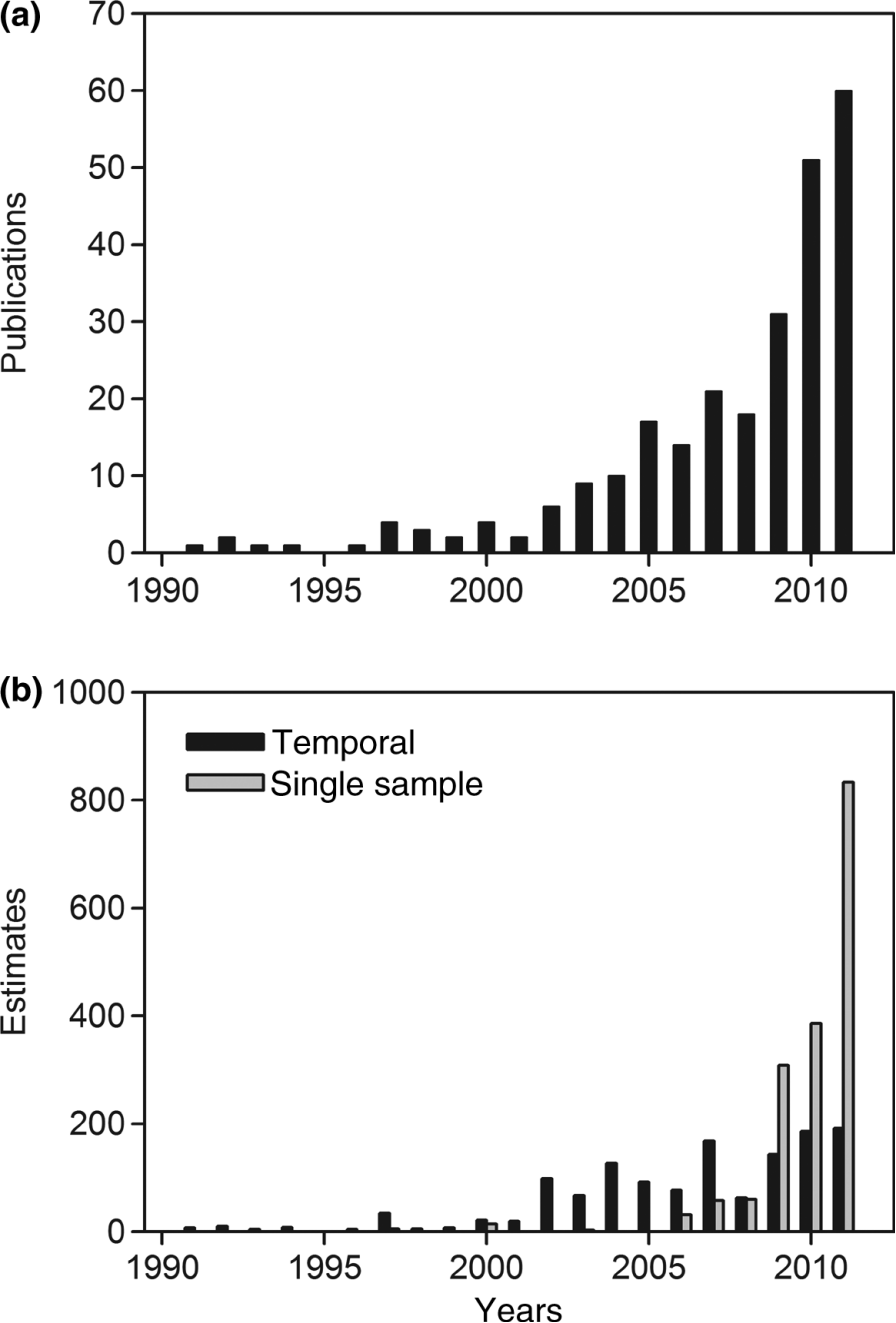
Annual trends of empirical studies on contemporary *N*_e_ based on genetic data. Given are (a) the number of *N*_e_ studies from 1990 to 2011 and (b) the number of published *N*_e_ estimates based on temporal methods and on single samples during the same time period. Data for 2012 are not shown as this year is still ongoing and therefore the summary of estimates is likely incomplete.

Our search located 2617 contemporary *N*_e_ estimates (1837 new estimates since 2008) published in 233 studies (151 new publications since 2008). A steady increase in publications reporting *N*_*e*_ estimates has occurred over the past 20 years (Fig. 1a), since empirical methods for estimating contemporary *N*_e_ started being applied using genetic data. Recent years have seen the development (Tallmon et al. 2008; Wang 2009) or refinement (Waples and Do 2008) of these methods using single samples, which affords the practical estimation of *N*_*e*_ based on a random sample of genotyped individuals (Hill 1981). This is reflected in the growing use of single sample approaches relative to temporal methods that require at least two samples separated usually by multiple generations (Fig. 1b). An important distinction is whether estimates generated from these approaches reflect *N*_e_ or the effective number of breeders (*N*_b_), two properties that are not equal but frequently confused (Table 1). We treat and discuss *N*_e_ and *N*_b_ separately whenever appropriate.

**Table 1:** Overview of relevant population parameters and their definitions (and the abbreviation symbols used to refer to them in this manuscript). References provided whenever possible and relevant

Parameter	Symbol	Definition	References
Annual census population size	*N*_a_	The number of reproductively mature individuals in a population that may reproduce and hence contribute to the cohort of individuals born in that year. Not to be confused with (i) total annual census population size (adults and juveniles) and (ii) annual census population size based on breeders, nonbreeders, and senescents	Frankham (1995)
Arithmetic mean		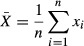	
Cohort		A group of individuals born in a given year, thus having the same age	Caswell (2001)
Effective population size	*N*_e_	The size of an ideal population experiencing the same rate of random genetic change over time as the real population under consideration. For the purpose of this review, we limit ourselves to contemporary effective population size	Wright (1931), Wright (1938)
Effective number of breeders	*N*_b_	The effective number of breeders contributing to a sample of offspring. When this offspring sample constitutes one single cohort, then *N*_b_ represents the effective number of breeders in a given year	Waples & Teel (1990)
Generation length		The average age of parents in the population, i.e., the reproductive output weighted by the age distribution of the parents.	Felsenstein (1971)
Harmonic mean		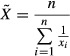	

### Trends in *N*_e_/*N* estimates

Only 28% of published studies with *N*_e_ estimates (66 of 233 publications) have explicitly attempted to link *N*_e_ to *N*. The 508 *N*_e_/*N* estimates reported in these 66 studies (240, or 47% of 508, new estimates since 2008, Table S1) comprise about 20% of all published *N*_e_ estimates using genetic data and these are analyzed in detail below.

### Correctly linked *N*_e_/*N* ratios

A considerable number of *N*_e_/*N* ratios reported to date have improperly linked *N*_e_ to *N*, despite the existence of guiding literature (Waples 2005). The relationship between *N*_e_ and *N* depends on both the nature of samples analyzed and the age structure of the population studied. Single sample methods based on linkage disequilibrium (Hill 1981; Waples and Do 2008) and relatedness (Wang 2009) estimate the number of adults that produced the sample, so when a population has discrete generations, this estimate applies to *N* in the previous generation. Temporal methods (e.g., Waples 1989; Wang and Whitlock 2003) generally apply to the harmonic mean generational *N* during the period delimited between the two samples (Kalinowski and Waples 2002). The important detail here is that the most recent generational *N* is *not* included in this calculation, for it has not yet been introduced to genetic drift. This situation becomes more complex in species with overlapping generations, a point we will return to later. For now, one important conclusion is that, regardless of the approach chosen to estimate *N*_e_, genetic and demographic data collected from exactly the same time period are not directly related (see also Nunney 1995). Hence, they are generally not compatible for the calculation of *N*_e_/*N*, unless one can explicitly assume that population size has been constant. Such an assumption is unlikely to be commonly justified in empirical studies of contemporary *N*_e_, as these are typically motivated by drastic declines in abundances of the study species (e.g., Ardren and Kapuscinski 2003; Johnson et al. 2004; Alo and Turner 2005; Fraser et al. 2007b; Henry et al. 2009; Riccioni et al. 2010; Zschokke et al. 2011). Under such circumstances, the untenable assumption of constant population size is most likely to yield upwardly biased *N*_e_/*N* ratios.

Using the recommendations of Waples (2005), we found that 31% of reported *N*_e_/*N* ratios (160 of 508 estimates) can be presumed free of bias caused by improper linking between *N*_e_ and *N*. These 160 estimates are roughly equally divided into estimates of *N*_b_/*N*_a_ and *N*_e_/*N*, where *N*_a_ is the adult census population size in a given year (Table 1). After further accounting for age structure in *N*_*e*_ estimation using temporal methods, only slightly over half of these *N*_e_/*N* ratio estimates remain, which is less than 4% of all published *N*_e_ estimates (93 of 2617).

### Degree of uncertainty in *N*_e_/*N* ratios and implications for inferring *N*_e_/*N* ratios

**Figure 2:**
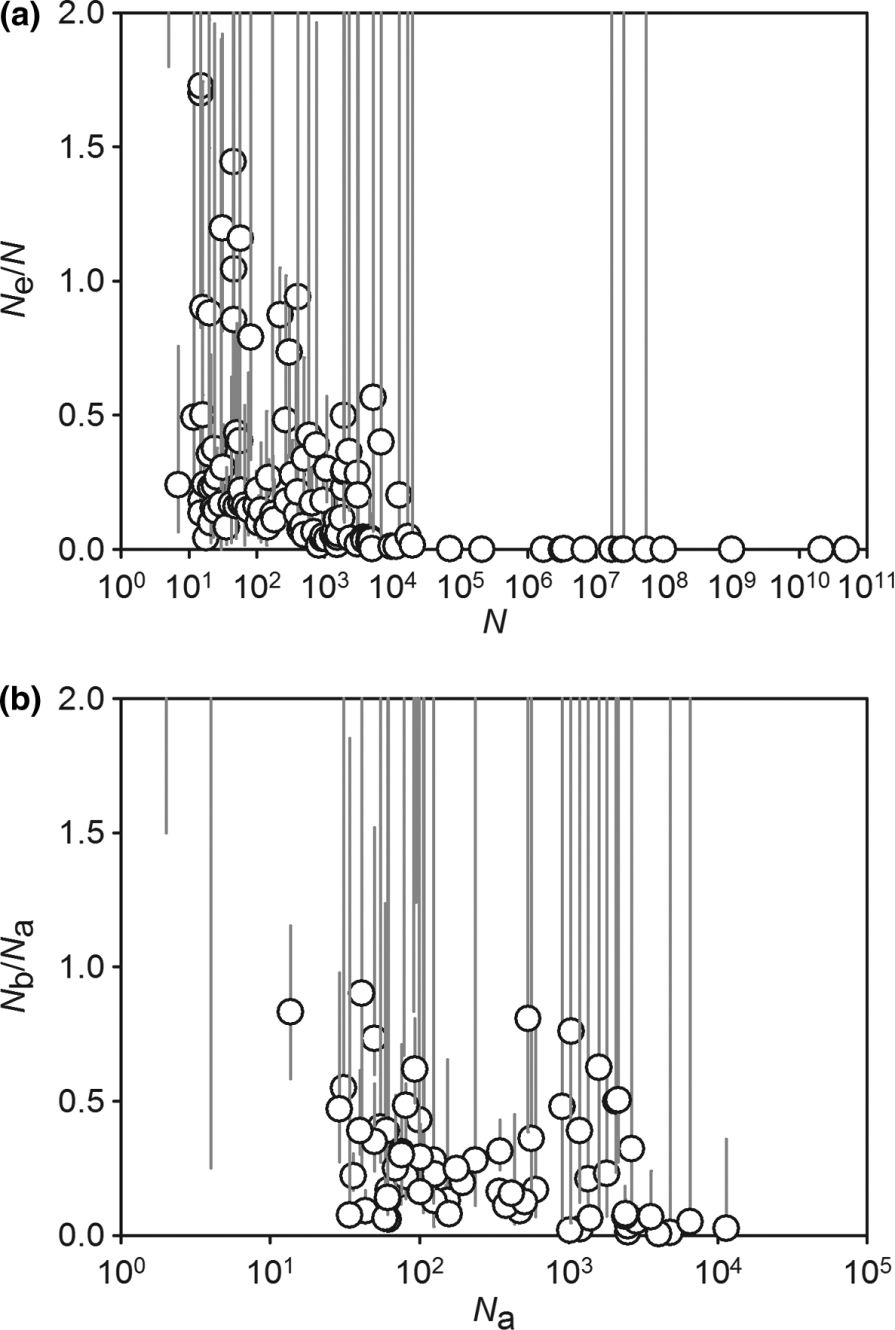
Uncertainty in estimates of the ratio of (a) *N*_e_ to adult census population size (*N*) and (b) *N*_b_ to annual census population size (*N*_a_), quantified by including the 95% confidence intervals surrounding *N*_e_ or *N*_b_ estimates, respectively. Note that some point estimates of these ratios where much larger than 2.0, but the *y*-axis scales were not extended to avoid blurring any trends at lower values.

Even if *N*_e_ and *N* are correctly linked, both parameters need to be estimated with accuracy and precision. However, our survey suggests that uncertainty in *N*_e_ or *N* estimates (e.g., 95% confidence intervals [CI] or credible regions) has been insufficiently translated explicitly into uncertainty in *N*_e_/*N* ratios. For example, after accounting for uncertainty in *N*_e_, plots of 95% CI for *N*_e_ versus *N* show that these often range anywhere from nearly zero to 1 (Fig. 2a). In fact, 31% of estimated *N*_e_/*N* ratios overlap with 1, of which more than half also reach below *N*_e_/*N* = 0.01. A similar result is obtained when considering the ratio between *N*_b_ estimates and annual census population size (*N*_a_) (Fig. 2b; see also Table 1), with 45% of *N*_b_/*N*_a_ ratios overlapping with 1 (11% of which also reach below *N*_b_*/N*_a_ = 0.01).The uncertainty in *N*_e_*/N* ratios thus frequently spans a minimum of two orders of magnitude.

A similar trend is observed when accounting for uncertainty in *N* estimates, even when *N*_*e*_ estimates are precise and accurate. We could only locate six empirical studies that reported CI for *N* (Jehle et al. 2001, 2005; Miller and Waits 2003; Charlier et al. 2011; Belmar-Lucero et al. 2012, Moyer et al. 2012). Of the 41 *N*_e_/*N* or *N*_b_*/N*_a_ ratios reported in these studies, 67% of comparisons contained an *N*_e_ estimate significantly smaller than the estimate of *N* (i.e., the 95% CI for the two parameters did not overlap), but this is just 5.5% (28 of 508) of all *N*_e_/*N* estimates and only 1.1% of all published *N*_e_ estimates.

Unfortunately, the challenge of incorporating a known (and the likely large) variance in *N* into the variance of *N*_e_/*N* has so far received scant attention in the literature. Possibly this situation could be improved in the future with the application of the Delta method (Oehlert 1992).

### So what are ‘typical’, correctly linked *N*_e_/*N* ratios?

In light of the inherent imprecision often surrounding *N*_e_ and/or *N* estimates, given that previous assessments of *N*_e_*/N* ratios (Palstra and Ruzzante 2008) did not account for improper linking of *N*_e_ to *N*, and given the large amount of new estimates published, we think it is justified to revisit this question with the present data. Available data on correctly linked *N*_e_/*N* ratios include 31 *N*_e_/*N* estimates and 62 *N*_b_/*N*_a_ estimates, where median values for *N*_e_/*N* and *N*_b_/*N*_a_ ratios were found to be 0.231 and 0.225, respectively. These median values are higher than previously reported values of 0.14 for genetic (Palstra and Ruzzante 2008) and 0.11 for mainly demographic *N*_e_ estimates (Frankham 1995). Hence, correctly linking the two parameters might increase the general magnitude of the *N*_e_/*N* ratio by a factor two (see also Nunney 1995) and bring them closer to theoretically expected values (Nunney 1993, 1996). We also observe that these values differ substantially from the medians based on all available estimates (*N*_e_/*N* = 0.123 and *N*_b_/*N*_a_ = 0.163) and analyses of variance indicate that the former are also more precise (despite being based on far fewer data points). Overall, although tempting as it may be to make a statement about the general magnitude of *N*_e_/*N* for natural populations, we necessarily reiterate, as have others in the past (Frankham 1995; Palstra and Ruzzante 2008), that our estimated *N*_e_/*N* medians should be interpreted with extreme caution: their taxonomic coverage is limited (based on fishes, amphibians, and insects only) and their range is considerable (0.01–0.95). Furthermore, our data plots combine all taxa and there are good reasons to suspect that *N*_e_/*N* ratios will differ among populations within species, among related species, and among different taxonomic groups, especially those characterized by different life history survival curves (cf. Palstra and Ruzzante (2008)).

### Is there a relationship between *N*_e_ and *N*?

**Figure 3:**
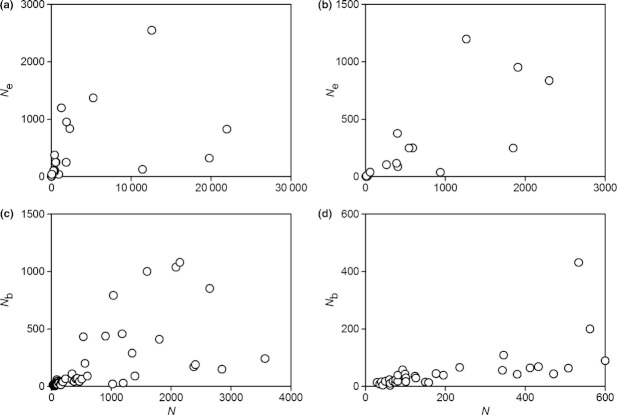
Relationships between (a) effective population size (*N*_e_) and generational census size and (c) effective number of breeders (*N*_b_) and annual census size based on the subset of empirical estimates that were correctly linked and free of bias due to age structure. For clarification, the same data are also displayed at smaller scales (b,d).

This is certainly a relevant question to explore because if simple conversions exist between *N*_e_ and *N*, limited conservation resources could be saved on the estimation of one variable to infer both, as pointed out in a recent review (Luikart et al. 2010). We therefore regressed the two parameters using only those data points that were correctly linked and unbiased due to age structure. Figure 3a,c shows that no simple linear relationship exists between estimates of *N*_e_ and *N* or *N*_b_ and *N*_a_ (simple linear regression, *r*^2^ = 0.11, *P* = 0.556, *r*^2^ = 0.05, *P* = 0.739, respectively). Interestingly, log-linear relationships are a better fit for both data sets (*N*_e_ vs. *N*, *r*^2^ = 0.43, *P* = 0.019; *N*_b_ vs. *N*_a_, *r*^2^ = 0.21, *P* = 0.063), suggesting that a positive, albeit variable, relationship between *N*_e_ and *N* may only exist at (very) low abundances (Fig. 3b,d). Moreover, correlation coefficients were always lower for regression analyses based on all data points (results not shown), which encouragingly suggests that additional correctly linked *N*_e_/*N* (and *N*_b_/*N*_a_) ratios in future studies could enhance our understanding of these ratios for natural populations. Naturally, these analyses ignore the large variation in life history that is contained in the database, which may have weakened any real biological relationships present in species with similar life histories. Nevertheless, our quantitative survey underscores that until similar surveys are conducted in the future with the addition of substantially more *N*_e_/*N* data, researchers should be extremely cautious when making inferences about *N*_e_ based on *N*, and vice versa.

## Recommendations and considerations

Our compendium and appraisal contains two salient conclusions. First, there is a need to better report uncertainty in both *N*_e_ and *N*, but particularly the latter, in studies linking *N*_e_ to *N*. Second, more attention needs to be paid to correctly linking *N*_e_ and *N*. How to do this was not well understood before Waples's (2005) criteria and since then, correctly linked *N*_e_/*N* ratios have encouragingly increased from 14.8% (26 of 179 estimates) to 38.4% (126 of 328 estimates). Yet, this final value suggests that researchers should continue to pay meticulous attention to the issue.

The overall lower scrutiny applied to *N* estimation in the same studies that estimate *N*_e_ is probably due to a number of common factors relating to the difficulty in estimating *N* in organisms: (i) characterized by secretive or obscure behaviors; (ii) inhabiting environments that make conducting population censuses challenging; and/or importantly (iii) having overlapping generations or repeat breeding (iteroparity). The ratio *N*_e_/*N* obviously depends on which definition of *N* is used (Nunney and Elam 1994). Here, we propose that, where feasible, *N* should reflect the number of reproductively mature adults in a population, as it is their ecology and reproductive biology that principally shape *N*_e_ (Frankham 1995). For semelparous species, these calculations are relatively straightforward and have, for example, helped elucidating the effects of variance in recruitment and population growth rate on the *N*_e_/*N* ratio (Waples 2002; Waples et al. 2010).

For iteroparous species with overlapping generations, some of the challenges related to calculating *N*_e_/*N* may be overcome through careful a priori consideration of the sampling design. It is often much easier to census adults breeding in a given season, and a single cohort sample applies to just such a property. A drawback is that these estimates will reflect the annual effective breeder size (*N*_b_) and much still remains to be understood on how this parameter relates to *N*_e_ (see Waples 1990, Waples et al. 2011). Hence, more empirical genetic studies that explicitly compare *N*_b_ and *N*_e_ will be needed. For example, genetic monitoring should facilitate sampling designs of several consecutive cohorts to estimate *N*_b_ using single sample approaches, with consecutive cohorts being analyzed jointly to estimate *N*_e_ using a temporal cohort model (Jorde and Ryman 1995; Jorde 2012). Alternately, researchers could analyze samples that aim to characterize the genetic make-up of an entire generation length, by pooling several annual samples of mixed cohorts (e.g., Palstra et al. 2009).

Finally, life table analyses (reviewed in Caswell 2001) continue to be an exception rather than the rule in empirical genetic studies of *N*_e_. This is unfortunate, as they can be used to directly estimate both *N* and *N*_e_ (Age *N*_e_, Waples et al. 2011) as well as provide demographic parameters to genetically estimate *N*_e_ using the cohort model. They will facilitate the interpretation of empirical *N*_*e*_ estimates in the context of population dynamics and species biology and, importantly, aid in the formulation of management recommendations.

Where the challenges highlighted above in estimating *N*_e_ for semelparous and iteroparous species can be practically overcome (reviewed or detailed in Jorde and Ryman 1995; Waples 2005; Palstra et al. 2009; Wang 2009; Waples et al. 2010, 2011), we note that there is an extensive, century-old literature on estimation of *N*, predominantly through the use of various mark-and-recapture methods (Ricker 1975; Seber 1982; Pollack et al. 1990). We do not review this rich literature here, but as in the estimation of *N*_e_, we strongly urge authors to carefully consider the sampling assumptions underlying the estimation of *N* more explicitly in the future when linking *N*_e_ and *N*. Researchers should also (i) provide more details on the methods used to calculate *N*, (ii) report the measure of uncertainty surrounding *N* estimates, and (iii) distinguish whether *N* is based on only adult breeders or breeders and senescent individuals (see Table 1). An excellent review (Luikart et al. 2010) also exists on how molecular genetic data may be utilized to noninvasively estimate *N* for species where it is impossible or harmful to handle the number of individuals required for traditional estimation.

The reality though is that some of the issues highlighted above (i.e., the difficulties in estimating *N* or *N*_e_ due to overlapping generations), and others, such as linking *N*_e_ to *N* in iteroparous species, may not be easily overcome without the use of considerable resources (multiyear field work and genotyping, parentage analysis, etc.). Whether this is deemed a major concern in a given situation may depend on the research question, the study system, or how large *N*_e_ and *N* are likely to be. For instance, if the goal of the research is to compare populations over several orders of magnitude of size (*N*_e_, *N*), it may not be that problematic if *N*_e_/*N* ratios are off by an order of magnitude due to incorrect linking or estimation of either *N*_e_ or *N*, provided that the assumptions are acknowledged and the errors are proportional across all populations sampled (e.g., Belmar-Lucero et al. 2012). Conversely, if the conservation fate of a species or population is being interpreted through *N*_e_ and/or *N* data, great caution relating to uncertainty is warranted. For example, in salmonid fishes, a group of related, socioeconomically important species for which the most *N*_e_/*N* estimates were available (*n* = 98, of which 65 were independent, whether free of bias or not), the range of *N*_e_/*N* estimates across populations within five species had a fourfold to 100-fold difference (Table S2). Clearly, such variation could translate into vastly different conservation implications when using one variable to infer the magnitude of the other (*N*_e_ from *N*, and vice versa). Overall, our hope in raising these issues here is to stimulate further discussion on such important topics in the future of conservation genetics in general, and of *N*_e_/*N* estimation in particular.

## Conclusion

There is now an extensive set of genetic tools available for estimating *N*_e_ (Waples 1989; Beaumont 2003; Wang and Whitlock 2003; Leberg 2005; Wang 2005, 2009; Jorde and Ryman 2007; Tallmon et al. 2008; Waples and Do 2008, 2010; Luikart et al. 2010). Encouragingly, the 2617 *N*_e_ estimates from the 233 studies we could locate suggest that empirical researchers are taking full advantage of these approaches. However, our quantitative survey suggests that research into *N*_e_ estimation could place a stronger focus on simultaneously estimating and correctly linking *N*_e_ and *N* as an additional step. This will stimulate considerations of *N*_e_ and *N* in the broader conservation context and will facilitate a better understanding of the relative importance of the various stochastic and deterministic forces that shape population persistence (see below). Apart from the need for meticulous calculation of both *N*_e_ and *N*, we also suggest that several important research areas will be enriched from doing so, both for new and expert researchers alike.

Some of these research topics have been reviewed or discussed in other, recent papers, such as understanding (i) the range and conditions over which *N*_e_/*N* can be assumed to be constant within populations (Vucetich et al. 1997; Waples 2005); (ii) the biological plausibility of genetic compensation or other factors that might lead to shifting *N*_e_/*N* ratios within populations (Ardren and Kapuscinski 2003; Fraser et al. 2007b; Watts et al. 2007); (iii) the variation in *N*_*e*_/*N* ratios across populations within species (Wright 1938; Frankham 1995; Shrimpton and Heath 2003; Palstra and Ruzzante 2008; Luikart et al. 2010; Belmar-Lucero et al. 2012); (iv) the role that life history plays in affecting the *N*_e_/*N* ratio in species (Lee et al. 2011), particularly for species with extremely low *N*_e_/*N* ratios such as marine fishes (e.g., Hauser et al. 2002; Turner et al. 2002); and (v) the likely possibility that *N*_e_/*N* is reduced by multiple factors which can act in tandem, whether due to interactions between population size and/or variance in reproductive success, reproductive biology, or anthropogenic pressures such as fisheries-induced size-selective mortality (Therkildsen et al. 2010; Lee et al. 2011; Belmar-Lucero et al. 2012).

Finally, some research topics are just emerging and therefore demand further investigation. For example, we still know little about how demographic (*N*) and evolutionary potential (*N*_e_) can feedback on one another within populations. Factors facilitating positive population growth at low *N*, and hence long-term viability, can result in a few individuals contributing disproportionately to the next generation in genetic terms, reducing *N*_e_ (Lee et al. 2011). In another case, reduced *N*_e_/*N* associated with a more complex age structure was found to actually confer greater resilience to environmental stochasticity (Gaggiotti and Vetter 1999). Whether such trade-offs are sufficiently strong to affect evolutionary potential awaits further empirical investigation but their recognition may help to guide the balancing of demographic and genetic goals in conservation.

## Supporting Information

**Table S1.** Overview of publications with empirical Ne/N ratio estimates on wild populations.

**Table S2.** Variability of empirical Ne/N ratio estimates within wild populations of salmonid fish species.
