# Supplementary material for: Effective/census population size ratio estimation: a compendium and appraisal
Source: Ecol Evol. 2012 Jul 25;2(9):2357–65. doi: 10.1002/ece3.329 (PMC3488685; doi:10.1002/ece3.329)
Supplement: Supplementary file 1 [file ece30002-2357-SD1.pdf]

Publication	*Species*	N_b/N_a	N_e/N
Husband & Barrret (1992)	*Echhornia paniculata*		10
Ingvarsson & Olsson (1997)	*Phalacrus substriatus*		15
Miller & Kapuscinki (1997)	*Esox lucius*		4
Scribner et al (1997)	*Bufo bufo*	18	
Jehle et al (2001)	*Triturus marmoratus*	7	5
Hauser et al (2002)	*Pagrus auratus*		9
Heath et al (2002)	*Oncorhynchus mykiss*		9
Turner et al (2002)	*Scaeniops ocellatus*		2
Ardren & Kapuscinski (2003)	*Oncorhynchus mykiss*	6	
Hutchinson et al (2003)	*Gadus morhua*		1
Miller & Waits (2003)	*Ursus arctos*		4
Shrimpton & Heath (2003)	*Oncorhynchus tsawytscha*		13
Borkowska & Ratkiewicz (2004)	*Chletrionomys glareolus*		2
Johnson et al (2004)	*Tympanuchus cupido*		28
Kaeuffer et al (2004)	*Felis catus*		4
Rowe & Beebee (2004)	*Bufo calamita*		6
Tallmon et al (2004)	*Ursus arctos*		1
Aguilar et al (2005)	*Esox lucius*		2
Alo & Turner (2005)	*Hybognathus amarus*		4
Consuegra et al (2005)	*Salmo salar*		16
Hoarau et al (2005)	*Pleuronectes platessa*		2
Jehle et al (2005)	*Triturus marmoratus*		10
Spidle et al (2005)	*Salmo salar*		1
Brede & Beebee (2006)	*Rana temporaria*	11	
Epps et al (2006)	*Ovis canadensis nelsoni*		23
Gomez-Uchida & Banks (2006)	*Sebastes crameri*		1
Lippe et al (2006)	*Moxostoma hubbsi*		3
Turner et al (2006)	*Hybognathus amarus*		6
Brown et al (2007)	*Falco peregrinus*		1
Fraser et al (2007)	*Salmo salar*		12
Hedgecock et al (2007)	*Ostraea edulis*	1	
Holycross & Douglas (2007)	*Crotalus willardi*		1
Kaueffer et al (2007)	*Ovis aries*		3
Schmeller & Merila (2007)	*Rana temporaria*	4	8
Watts et al (2007)	*Coenagrion mercuriale*		15
Bjorklund & Arrendal (2008)	*Lutra lutra*		1
Coyer et al (2008)	*Fucus serratus*		2
Zeller et al (2008)	*Eudiaptomus gracilis*		6
Beebee (2009)	*Bufo calamita*	35	
Bishop et al (2009)	*Crocodylus niloticus*	1	2
Cronin et al (2009)	*Ursus maritimus*		2
Franckowiak et al (2009)	*Sander vitreus*	7	20
Henry et al (2009)	*Panthera tigris altaica*		2
Palstra et al (2009)	*Salmo salar*	15	13
Portnoy et al (2009)	*Carcharhinus plumbeus*	3	2
Small et al (2009)	*Oncorhynchus keta*		16
Ursenbacher et al (2009)	*Trochulus caelatus*		7
Ursenbacher et al (2009)b	*Vipera berus*		6
Ficetola et al (2010)	*Rana latastei*	8	
Martin (2010)	*Cyprinodon nevadensis*		8
Phillipsen et al (2010)	*Rana pretiosa*	8	
Randall et al (2010)	*Canis simensis*		3
Watts et al (2010)	*Pleuronecta olatessa*		4
Brekke et al (2011)	*Notiomystis cincta*		6
Charlier et al (2011)	*Salmo trutta*		6
Cuveliers et al (2011)	*Solea solea*		7
Kanno et al (2011)	*Salvelinus fontinalis*		2
Kettle et al (2011)	*Araucaria nemorosa*		3
Nielsen et al (2011)	*Oncorhynchus mykiss*		3
Van Doornik et al (2011)	*Oncorhynchus tshawytscha*	16	
Grueber & Jamieson (2011)	*Porphyrio hochstetteri*		1
Pruett et al (2011)	*Tympanuchus pallidicinctus*		1
Belmar-Lucero et al (2012)	*Salvelinus fontinalis*		4
DeHaan et al (2012)	*Oregonichthys crameri*		6
Marsden et al (2012)	*Lycaon pictus*		10
Moyer et al (2012)	*Acipenser oxyrinchus oxyrinchus*	4	
