# Supplementary figures and images for: Effective/census population size ratio estimation: a compendium and appraisal
Source: Ecol Evol. 2012 Jul 25;2(9):2357–65. doi: 10.1002/ece3.329 (PMC3488685; doi:10.1002/ece3.329)

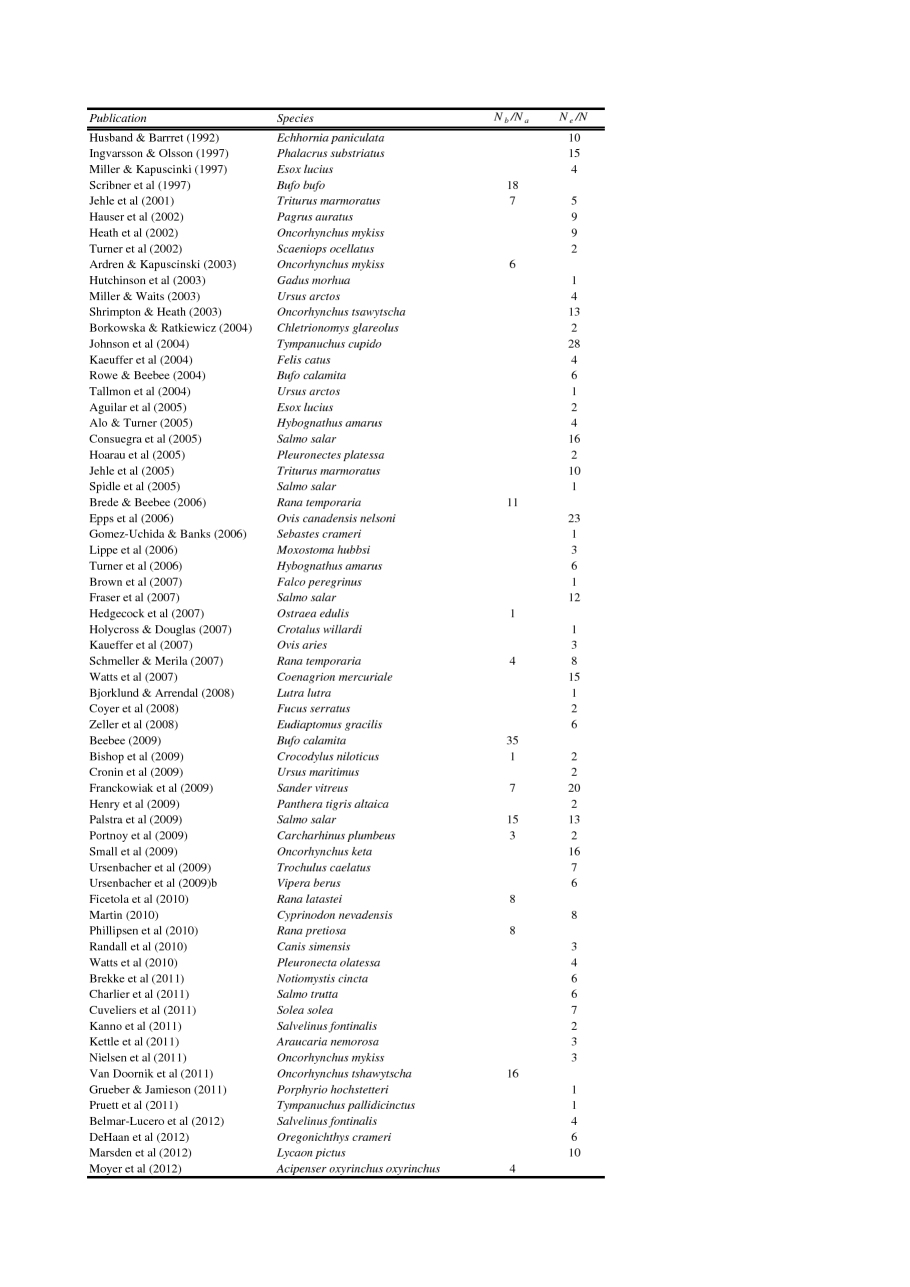

Supplement: Supplementary file 2 [file ece30002-2357-SD3.png]

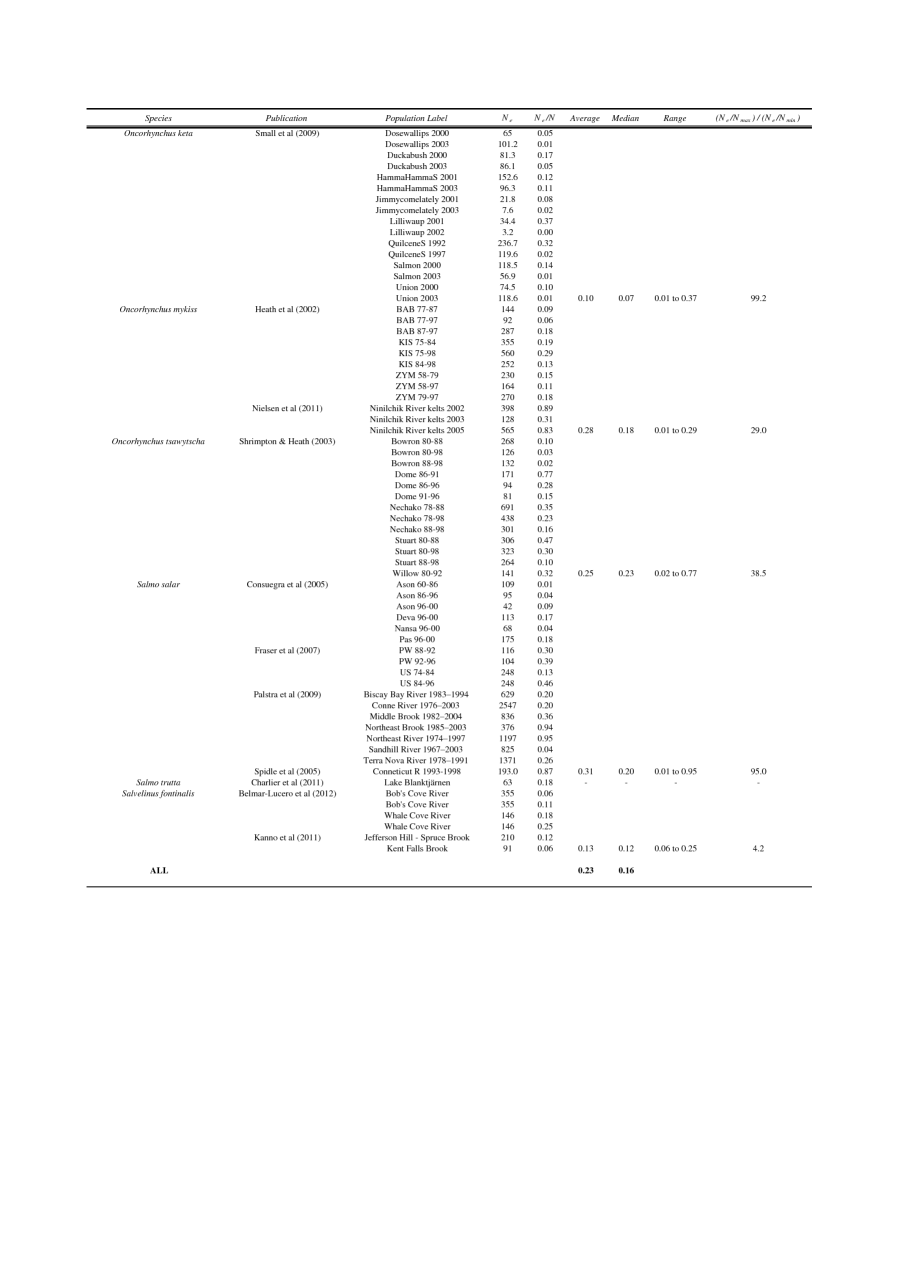

Supplement: Supplementary file 4 [file ece30002-2357-SD4.png]
