# Supplementary material for: Effective/census population size ratio estimation: a compendium and appraisal
Source: Ecol Evol. 2012 Jul 25;2(9):2357–65. doi: 10.1002/ece3.329 (PMC3488685; doi:10.1002/ece3.329)
Supplement: Supplementary file 3 [file ece30002-2357-SD2.pdf]

Species	*Publication*	*Population Label*	N_e	N_e/N	*Average*	*Median*	*Range*	$(N_e/N_{max})/(N_e/N_{min})$
Oncorhynchus keta	Small et al (2009)	Dosewallips 2000	65	0.05				
		Dosewallips 2003	101.2	0.01				
		Duckabush 2000	81.3	0.17				
		Duckabush 2003	86.1	0.05				
		HammaHammaS 2001	152.6	0.12				
		HammaHammaS 2003	96.3	0.11				
		Jimmycomelately 2001	21.8	0.08				
		Jimmycomelately 2003	7.6	0.02				
		Lilliwaup 2001	34.4	0.37				
		Lilliwaup 2002	3.2	0.00				
		QuilceneS 1992	236.7	0.32				
		QuilceneS 1997	119.6	0.02				
		Salmon 2000	118.5	0.14				
		Salmon 2003	56.9	0.01				
		Union 2000	74.5	0.10				
		Union 2003	118.6	0.01	0.10	0.07	0.01 to 0.37	99.2
Oncorhynchus mykiss	Heath et al (2002)	BAB 77-87	144	0.09				
		BAB 77-97	92	0.06				
		BAB 87-97	287	0.18				
		KIS 75-84	355	0.19				
		KIS 75-98	560	0.29				
		KIS 84-98	252	0.13				
		ZYM 58-79	230	0.15				
		ZYM 58-97	164	0.11				
		ZYM 79-97	270	0.18				
	Nielsen et al (2011)	Ninilchik River kelts 2002	398	0.89				
		Ninilchik River kelts 2003	128	0.31				
		Ninilchik River kelts 2005	565	0.83	0.28	0.18	0.01 to 0.29	29.0
Oncorhynchus tsawytscha	Shrimpton & Heath (2003)	Bowron 80-88	268	0.10				
		Bowron 80-98	126	0.03				
		Bowron 88-98	132	0.02				
		Dome 86-91	171	0.77				
		Dome 86-96	94	0.28				
		Dome 91-96	81	0.15				
		Nechako 78-88	691	0.35				
		Nechako 78-98	438	0.23				
		Nechako 88-98	301	0.16				
		Stuart 80-88	306	0.47				
		Stuart 80-98	323	0.30				
		Stuart 88-98	264	0.10				
		Willow 80-92	141	0.32	0.25	0.23	0.02 to 0.77	38.5
Salmo salar	Consuegra et al (2005)	Ason 60-86	109	0.01				
		Ason 86-96	95	0.04				
		Ason 96-00	42	0.09				
		Deva 96-00	113	0.17				
		Nansa 96-00	68	0.04				
		Pas 96-00	175	0.18				
	Fraser et al (2007)	PW 88-92	116	0.30				
		PW 92-96	104	0.39				
		US 74-84	248	0.13				
		US 84-96	248	0.46				
	Palstra et al (2009)	Biscay Bay River 1983–1994	629	0.20				
		Conne River 1976–2003	2547	0.20				
		Middle Brook 1982–2004	836	0.36				
		Northeast Brook 1985–2003	376	0.94				
		Northeast River 1974–1997	1197	0.95				
		Sandhill River 1967–2003	825	0.04				
		Terra Nova River 1978–1991	1371	0.26				
	Spidle et al (2005)	Conneticut R 1993-1998	193.0	0.87	0.31	0.20	0.01 to 0.95	95.0
Salmo trutta	Charlier et al (2011)	Lake Blanktjärnen	63	0.18	-	-	-	-
Salvelinus fontinalis	Belmar-Lucero et al (2012)	Bob's Cove River	355	0.06				
		Bob's Cove River	355	0.11				
		Whale Cove River	146	0.18				
		Whale Cove River	146	0.25				
	Kanno et al (2011)	Jefferson Hill - Spruce Brook	210	0.12				
		Kent Falls Brook	91	0.06	0.13	0.12	0.06 to 0.25	4.2
**ALL**					**0.23**	**0.16**		
